# Effective Governing Equations for Viscoelastic Composites

**DOI:** 10.3390/ma16144944

**Published:** 2023-07-11

**Authors:** Laura Miller, Ariel Ramírez-Torres, Reinaldo Rodríguez-Ramos, Raimondo Penta

**Affiliations:** 1 School of Mathematics & Statistics, University of Glasgow, Glasgow G12 8QQ, UK; 2 Facultad de Matemática y Computación, Universidad de La Habana, La Habana 10400, Cuba; 3 PPG-MCCT, Universidade Federal Fluminense, Av. dos Trabalhadores 420, Vila Sta. Cecília, Volta Redonda 27225-125, RJ, Brazil

**Keywords:** homogenization, viscoelasticity, fluid–structure interaction

## Abstract

We derive the governing equations for the overall behaviour of linear viscoelastic composites comprising two families of elastic inclusions, subphases and/or fibres, and an incompressible Newtonian fluid interacting with the solid phases at the microscale. We assume that the distance between each of the subphases is very small in comparison to the length of the whole material (the macroscale). We can exploit this sharp scale separation and apply the asymptotic (periodic) homogenization method (AHM) which decouples spatial scales and leads to the derivation of the new homogenised model. It does this via upscaling the fluid–structure interaction problem that arises between the multiple elastic phases and the fluid. As we do not assume that the fluid flow is characterised by a parabolic profile, the new macroscale model, which consists of partial differential equations, is of Kelvin–Voigt viscoelastic type (rather than poroelastic). The novel model has coefficients that encode the properties of the microstructure and are to be computed by solving a single local differential fluid–structure interaction (FSI) problem where the solid and the fluid phases are all present and described by the one problem. The model reduces to the case described by Burridge and Keller (1981) when there is only one elastic phase in contact with the fluid. This model is applicable when the distance between adjacent phases is smaller than the average radius of the fluid flowing in the pores, which can be the case for various highly heterogeneous systems encountered in real-world (e.g., biological, or geological) scenarios of interest.

## 1. Introduction

Materials can be described and classified in a number of ways. For example, viscous materials have a time-dependent deformation when subjected to a strain and also resist shear flow. Another example is elastic materials that will strain when a load is applied but will returned to the same unstressed state when the load is removed. Viscoelastic materials are characterised by having both an elastic and a viscous response under deformation, and therefore can be described as exhibiting time-dependent strain [1,2].

There are a large variety of physical settings where we have materials that exhibit a viscoelastic response. For example, in the human spine, under normal body weight, the disks get shorter with time, which means people are shorter in the evening, and lying down (removing the body weight) allows the disks to return to normal length by morning [3,4]. Human skin can also be described as viscoelastic and can be useful in diagnostic techniques and scar modelling [5,6,7]. The theory of viscoelasticity has also been used to consider materials that have a composite-like structure. Some examples of this are found in several biological contexts such as cortical and trabecular bone [8,9]. Viscoelastic composites including fibres have been very useful in engineering and manufacturing processes due to the fact that the properties can be optimised [10].

These viscoelastic systems are usually characterised by multiple physical scales of interest. They possess a fine scale structure and this is where the various solid and fluid interactions are clearly visible (the microscale). This scale is considerably smaller than the length associated with the complete viscoelastic material (the macroscale).

Viscoelastic composites have recently been addressed by a multiscale modelling approach. In [11,12,13], a variety of different methods have been used to incorporate the different microstructural information in an computationally feasible manner. The effective response of a material that is based upon the properties of the individual constituents can be described by micromechanical models. These properties can be the viscoelastic moduli or geometrical arrangement and volume fraction of the different microstructural constituents.

When addressing a multiscale system, there are a variety of approaches that can be taken to transform a fluid–structure interaction (FSI) problem into a complete macroscale governing system. These types of procedures can be categorised as *homogenization techniques*. Examples of such techniques are mixture theory, effective medium theory, volume averaging and asymptotic homogenization. The choice as to which method will be suitable for the system under study should be made depending on the application of the model and the information you wish to be encoded or available from the macroscale model; however, each approach has its own benefits.

The techniques including mixture theory and effective medium theory are micromechanical approaches. These are useful when it is desirable to obtain estimates of the poroelastic coefficients for particular pore geometries. These specific geometries include spherical, ellipsoidal, or penny-shaped or in the case of diluted pores. This was considered in [14]. Volume averaging techniques can be used when the goal is to derive the functional form of the equations governing the macroscale. When using this approach, the macroscale model coefficients are in general not encoding the microstructure of the material and require further data such as experimental results to obtain them. For a further summary and comparison of homogenization techniques, the reader is directed to [15,16].

Previously, the asymptotic homogenization method has been a popular approach to study poroelastic materials such as in [17,18,19]. This theory was then extended to consider the case of an inviscid fluid [20]. The method has also been employed to study elastic composites [21,22,23,24] and electroactive materials [25,26,27,28]. The technique has also previously been exploited to address problems in viscoelasticity. For example, the technique allowed for analytical closed form expressions for the effective coefficients of fibrous viscoelastic composites to be found in [29,30,31]. In [32], the authors study the homogenized properties of linear viscoelastic composite materials in three dimensions by means of a semi-analytical approach combining the asymptotic homogenization method (AHM) with numerical computations performed by finite element simulations. The work by [33] addresses the calculation of the effective properties of non-aging linear viscoelastic composite materials and investigates the effective creep and relaxation behaviour for a variety of fibre and inclusion reinforced structures.

In this manuscript, we apply the asymptotic homogenization method to upscale the FSI problem between two families of solid obstacles and a Newtonian fluid phase. In this case, the medium could not be considered a porous material. Both families of elastic subphases are in contact with the fluid and each other. We assume that the length scale at which the different solid phases and the fluid are clearly resolved can be compared with the distance between each of the phases which we call the microscale. We make the assumption that this scale is much smaller than the macroscale. We then carry out an upscaling on a system of equations that describe the behaviour of each phase and is closed by the continuity of stresses and displacements on the solid–solid interfaces, and the continuity of stresses and velocities on the fluid–solid interfaces. The application of the asymptotic homogenization method leads to a set of viscoelastic-type equations, which are a generalization of linear elastic composites [22] and those found in [17] in which the authors discussed the emergence of a viscoelastic model through the homogenization process of a composite material comprising a single solid phase and the fluid.

The novel model derived in this manuscript has coefficients that encode the properties of the microstructure and are to be computed by solving a single local differential FSI problem where the solid and the fluid phases are all present and described by the one problem. The new model has a key difference from formulations of previous viscoelastic models. That is, our model encodes multiple different elastic phases interacting with the fluid and each other, in comparison to some previous viscoelastic formulations such as [17] where there is only one fluid and one solid phase, or indeed in the case where the interactions between a solid viscoelastic phase and a fluid are considered [34,35]. The addition of the extra interactions between multiple phases to the model is particularly beneficial to physical applications such as in the bones [8,9] where there are a variety of different microscale constituents, such as collagen and minerals, and in articular cartilage [36,37]. The coefficients capture the differences in elastic and mechanical properties between the phases, as well as the discrepancies in the elastic properties at different points in the microstructure. The model can also be compared with that of poroelastic composites [38] where we see the important difference that the scaling of the fluid viscosity has. The macroscale model coefficients capture the geometry, elastic properties and interactions of the microscale constituents and are determined by solving a local differential FSI problem where the solid and the fluid phases are all present and described by the one problem.

We organise this paper in the following manner. Section 2 sees the introduction of the fluid–structure interaction problem describing the interactions occurring between the two families of elastic subphases and the fluid phase. We continue the development of the work by performing a multiple scales analysis of the FSI problem in Section 3. This leads to the derivation of the macroscale governing equations for the homogenized effective mechanical behaviour of viscoelastic composites. In Section 4, we present the macroscale model and compare the results here with previously known models in the literature such as [17,38]. In Section 5, the work concludes with discussions surrounding the limitations of the current model and further directions for future development.

## 2. The Fluid–Structure Interaction Problem

Our problem begins with a set $\Omega \in R^{3}$, where Ω comprises two families of *N* disjoint solid subphases $\Omega_{a}$ and $\Omega_{b}$ and an interconnected fluid domain $\Omega_{f}$. We have that

(1a) $$\begin{array}{cc} \; & \Omega_{a}=\overset{N}{\underset{\delta =1}{⋃}}\Omega_{\delta }, \end{array}$$

(1b) $$\begin{array}{cc} \; & \Omega_{b}=\overset{N}{\underset{\beta =1}{⋃}}\Omega_{\beta }. \end{array}$$

**Figure 1:**
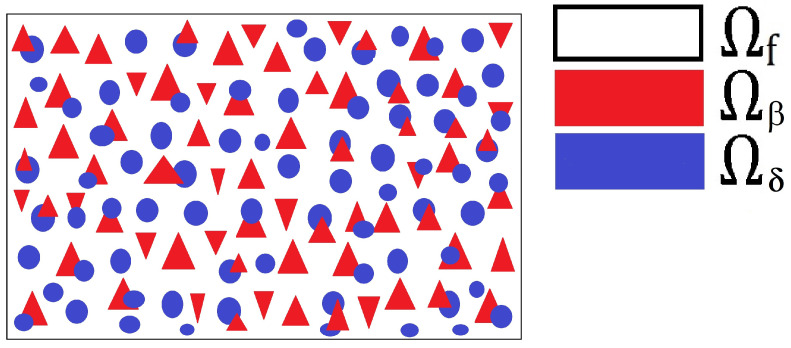
A 2D slice showing the different components of the 3D domain Ω. We indicate the fluid in white, and the two distinct families of subphases for all δ=1,…,N,andβ=1,…,N are shown in red and blue. We see that the fluid surrounds all the elastic phases and each elastic phases can either interact with other elastic phases or just the fluid.

We can write that the domain is $\bar{\Omega }={\bar{\Omega }}_{a}\cup {\bar{\Omega }}_{b}\cup {\bar{\Omega }}_{f}$. To illustrate this structure, we have provided Figure 1, which is a two-dimensional schematic of the domain Ω.

Before describing the equations that govern each of the domains in our structure we first wish to clarify the notation that will be used throughout this manuscript.

**Remark** **1** (Notation)**.** *We use the following for a generic field. For a scalar we use ordinary lowercase letters, e.g., v, for a vector we use boldface, e.g., v, and monospace font V is used for second rank tensor. We use uppercase calligraphic letters for third rank tensors, for instance V, and finally blackboard bold font V is used for fourth rank tensors.*

We first require a balance equation in each of the solid domains $\Omega_{\delta }$ and $\Omega_{\beta }$. We choose to neglect volume forces and inertia, so we can write for all δ=1,…,N,andβ=1,…,N

(2a) $$\begin{array}{cc} \; & \nabla \cdot T_{\delta }=0\;\mathrm{in}\;\Omega_{\delta }, \end{array}$$

(2b) $$\begin{array}{cc} \; & \nabla \cdot T_{\beta }=0\;\mathrm{in}\;\Omega_{\beta }. \end{array}$$

We use $T_{\delta }$ and $T_{\beta }$ as the solid stress tensors for each of the subphases $\Omega_{\delta }$ and $\Omega_{\beta }$, respectively. We make the choice that both families of subphases are general linear elastic solids. This means we can write the Cauchy stress tensors for $T_{\delta }$ and $T_{\beta }$ as

(3a) $$\begin{array}{cc} T_{\delta }= & C_{\delta }\nabla u_{\delta }, \end{array}$$

(3b) $$\begin{array}{cc} T_{\beta }= & C_{\beta }\nabla u_{\beta }, \end{array}$$

where we have that $u_{\delta }$ and $u_{\beta }$ denote the elastic displacement of each of the individual subphases from each family. The tensors $C_{\delta }$ and $C_{\beta }$ appearing in (3a)–(3b) are the fourth rank elasticity tensors in each subphase. These can be written in components as $C_{pqrs}^{\delta }$ and $C_{pqrs}^{\beta }$, for p,q,r,s=1,2,3. Each tensor $C_{\delta }$ and $C_{\beta }$ has right minor and major symmetries, these are

(4a) $$\begin{array}{cc} \; & C_{pqrs}^{\delta }=C_{pqsr}^{\delta };\;C_{pqrs}^{\beta }=C_{pqsr}^{\beta }, \end{array}$$

(4b) $$\begin{array}{cc} \; & C_{pqrs}^{\delta }=C_{rspq}^{\delta };\;C_{pqrs}^{\beta }=C_{rspq}^{\beta }, \end{array}$$

and the left minor symmetries can be obtain by combining (4a) and (4b). We can use the left minor symmetries to rewrite constitutive Equations (3a) and (3b) as

(5a) $$\begin{array}{cc} T_{\delta }= & C_{\delta }\xi \left(u_{\delta }\right), \end{array}$$

(5b) $$\begin{array}{cc} T_{\beta }= & C_{\beta }\xi \left(u_{\beta }\right), \end{array}$$

where we define

(6) $$\xi \left(•\right):=\frac{\nabla \left(•\right)+{\left(\nabla (•)\right)}^{T}}{2}$$

as the symmetric part of the gradient operator.

We also require a fluid balance equation, we can write

(7) $$\nabla \cdot T_{f}=0\;\mathrm{in}\;\Omega_{f},$$

with the stress tensor for the fluid, $T_{f}$. Our fluid can be described as incompressible and Newtonian, so therefore the constitutive law is

(8) $$T_{f}=-pI+2\mu \xi \left(v\right),$$

where we represent the viscosity fluid by μ the fluid velocity by v, and where *p* is the pressure. Since we have an incompressible fluid we require

(9) $$\nabla \cdot v=0.\;\mathrm{in}\;\Omega_{f},$$

Using the fluid constitutive law (8) in the fluid balance Equation (7) and together with the incompressibility constraint (9), this leads to the Stokes’ problem

(10) $$\mu \nabla^{2}v=\nabla p\;\mathrm{in}\;\Omega_{f}.$$

To have a complete FSI problem, we close it with appropriate interface conditions. These conditions are placed to describe the interactions between the fluid and the solid phases. The interfaces can be defined as follows: between the fluid and the δ-th inclusion/fibre/subphase we have $\Gamma_{\delta }:=\partial \Omega_{\delta }\cap \partial \Omega_{f}$; and between the fluid phase and the β inclusion/fibre/subphase as $\Gamma_{\beta }:=\partial \Omega_{\beta }\cap \partial \Omega_{f}$. Across each $\Gamma_{\delta }$ and $\Gamma_{\beta }$ we then enforce continuity of velocities and stresses, i.e.,

(11a) $$\begin{array}{cc} \;\;{\dot{u}}_{\delta }=v & \;\mathrm{on}\;\Gamma_{\delta }, \end{array}$$

(11b) $$\begin{array}{cc} T_{f}n_{\delta }=T_{\delta }n_{\delta } & \;\mathrm{on}\;\Gamma_{\delta }, \end{array}$$

(11c) $$\begin{array}{cc} \;\;{\dot{u}}_{\beta }=v & \;\mathrm{on}\;\Gamma_{\beta }, \end{array}$$

(11d) $$\begin{array}{cc} T_{f}n_{\beta }=T_{\beta }n_{\beta } & \;\mathrm{on}\;\Gamma_{\beta }, \end{array}$$

for all δ=1,…,N,andβ=1,…,N. We have used the notation ${\dot{u}}_{\delta }$ and ${\dot{u}}_{\beta }$ to describe the solid velocities in each inclusion/fibre/subphase $\Omega_{\delta }$ and $\Omega_{\beta }$, respectively. We must also note that $n_{\delta }$ and $n_{\beta }$ are the unit outward vectors that are normal to the interfaces $\Gamma_{\delta }$ and $\Gamma_{\beta }$. That is, these normals point into the fluid domain $\Omega_{f}$.

**Remark** **2** (Frequency Domain)**.** *In this work, we embrace the approach of [17], which means that we consider time harmonic motion. As such, a time dependent field, say φ(x,t), can be decomposed in a solely spatially varying component and a harmonic time variation, i.e., we assume that $\varphi \left(x,t\right)=\varphi_{0}\left(x\right)\exp\left(i\omega t\right)$. For instance, this then means that $\frac{\partial \varphi }{\partial t}=i\omega t$. In the remainder of this work, we will identify each field with their spatially varying component and omit the subscript ${}_{0}$ for the sake of simplicity of notation. This approach can be carried out without loss of generality as in principle the problem could be formulated in the frequency domain and so, in other words, every sufficiently smooth time dependency could be taken into account by spanning over the frequency domain by means of the Fourier transform operator.*

As such, assuming continuity of velocities at the interfaces, by means of Remark 2, we have

(12a) $$\begin{array}{cc} i\omega u_{\delta }=v & \;\mathrm{on}\;\Gamma_{\delta }, \end{array}$$

(12b) $$\begin{array}{cc} i\omega u_{\beta }=v & \;\mathrm{on}\;\Gamma_{\beta }. \end{array}$$

We also have the boundary between each of the different solid phases, which we write as $\Gamma_{\delta \beta }:=\partial \Omega_{\delta }\cap \partial \Omega_{b}$. We then impose continuity of stresses and displacements, which can be written as

(13a) $$\begin{array}{cc} T_{\delta }n_{\delta \beta }=T_{\beta }n_{\delta \beta } & \;\mathrm{on}\;\Gamma_{\delta \beta }. \end{array}$$

(13b) $$\begin{array}{cc} \;\;u_{\delta }=u_{\beta } & \;\mathrm{on}\;\Gamma_{\delta \beta }, \end{array}$$

for all δ=1…N,andβ=1,…,N. The unit vector $n_{\delta \beta }$ appearing in (13a) is normal to the interface $\Gamma_{\delta \beta }$ and is pointing into the inclusion $\Omega_{\delta }$.

We have now introduced all the equations necessary to carry out a multiple scales analysis. We do this by first non-dimensionalising the fluid–structure interaction problem we have just formed. This is done by introducing two distinct length scales. We use this sharp scale separation and apply the asymptotic homogenization method to the non-dimensionalised FSI problem. This leads to the derivation of the effective macroscale governing equations that describe the viscoelastic material.

## 3. Multiple Scales Analysis

We can now summarise the FSI problem we introduced in the previous section. We note that the fields in each of the equations here are all defined in the time domain and where the appearance of a time derivative is shown by the multiplication of the field by iω. We have

(14a) $$\begin{array}{cc} \nabla \cdot T_{\delta }=0\; & \;\mathrm{in}\;\Omega_{\delta }, \end{array}$$

(14b) $$\begin{array}{cc} \nabla \cdot T_{\beta }=0\; & \;\mathrm{in}\;\Omega_{\beta }, \end{array}$$

(14c) $$\begin{array}{cc} \nabla \cdot T_{f}=0\; & \;\mathrm{in}\;\Omega_{f}, \end{array}$$

(14d) $$\begin{array}{cc} \nabla \cdot v=0\; & \;\mathrm{in}\;\Omega_{f}, \end{array}$$

(14e) $$\begin{array}{cc} \;i\omega u_{\delta }=v & \;\mathrm{on}\;\Gamma_{\delta }, \end{array}$$

(14f) $$\begin{array}{cc} \;i\omega u_{\beta }=v & \;\mathrm{on}\;\Gamma_{\beta }, \end{array}$$

(14g) $$\begin{array}{cc} T_{f}n_{\delta }=T_{\delta }n_{\delta } & \;\mathrm{on}\;\Gamma_{\delta }, \end{array}$$

(14h) $$\begin{array}{cc} T_{f}n_{\beta }=T_{\beta }n_{\beta } & \;\mathrm{on}\;\Gamma_{\beta }, \end{array}$$

(14i) $$\begin{array}{cc} T_{\delta }n_{\delta \beta }=T_{\beta }n_{\delta \beta } & \;\mathrm{on}\;\Gamma_{\delta \beta }, \end{array}$$

(14j) $$\begin{array}{cc} \;\;u_{\delta }=u_{\beta } & \;\mathrm{on}\;\Gamma_{\delta \beta }. \end{array}$$

We can then use the constitutive relationships (5a), (5b), and (8), and the incompressibility constraint (14d), to rewrite the balance Equations (14a)–(14c) as

(15a) $$\begin{array}{cc} \nabla \cdot (C_{\delta }\xi \left(u_{\delta }\right))=0\; & \;\mathrm{in}\;\Omega_{\delta } \end{array}$$

(15b) $$\begin{array}{cc} \nabla \cdot (C_{\beta }\xi \left(u_{\beta }\right))=0\; & \;\mathrm{in}\;\Omega_{\beta } \end{array}$$

(15c) $$\begin{array}{cc} \;\;\;\mu \nabla^{2}v=\nabla p\; & \;\mathrm{in}\;\Omega_{f}, \end{array}$$

for all δ=1…N,andβ=1,…,N. To close the problem (14a)–(14j), we place appropriate external boundary conditions on ∂Ω.

### 3.1. Non-Dimensionalisation of the FSI Equations

As the system we are considering is multiscale, we identify two typical length scales. We first associate *L* with the size of the whole material Ω (*the macroscale*). The second scale of interest we denote with *d* and this refers to *the microscale*, which we assume can be compared to the distance between each of the subphases and the fluid. In order to clearly see the distinction between the scales, we carry out a non-dimensionalisation of the system (14a)–(14j). To perform the non-dimensional analysis, we assume that the system is characterised by a reference pressure gradient *C*, as well as the length scales to obtain

(16) $$\begin{array}{c} x=Lx^{\prime },\;C_{\delta }=CLC_{\delta }^{\prime },\;C_{\beta }=CLC_{\beta }^{\prime },\\ u_{\delta }=Lu_{\delta }^{\prime },\;u_{\beta }=Lu_{\beta }^{\prime },\;v=\frac{CL^{2}}{\mu }v^{\prime },\;p=CLp^{\prime }. \end{array}$$

It is possible to choose different scalings for the fluid velocity and these account for the effective behaviour of fluid flow in porous media [17,19,38,39].

We are able to use (16), and noticing that we can write

(17) $$\nabla =\frac{1}{L}\nabla^{\prime },$$

we obtain the non-dimensionalised fluid–structure interaction problem (14a)–(14j),

(18a) $$\begin{array}{cc} \nabla \cdot T_{\delta }=0\; & \;\mathrm{in}\;\Omega_{\delta } \end{array}$$

(18b) $$\begin{array}{cc} \nabla \cdot T_{\beta }=0\; & \;\mathrm{in}\;\Omega_{\beta } \end{array}$$

(18c) $$\begin{array}{cc} \nabla \cdot T_{f}=0\; & \;\mathrm{in}\;\Omega_{f} \end{array}$$

(18d) $$\begin{array}{cc} \nabla \cdot v=0\; & \;\mathrm{in}\;\Omega_{f} \end{array}$$

(18e) $$\begin{array}{cc} \;i\omega u_{\delta }=v & \;\mathrm{on}\;\Gamma_{\delta } \end{array}$$

(18f) $$\begin{array}{cc} \;i\omega u_{\beta }=v & \;\mathrm{on}\;\Gamma_{\beta } \end{array}$$

(18g) $$\begin{array}{cc} T_{f}n_{\delta }=T_{\delta }n_{\delta } & \;\mathrm{on}\;\Gamma_{\delta } \end{array}$$

(18h) $$\begin{array}{cc} T_{f}n_{\beta }=T_{\beta }n_{\beta } & \;\mathrm{on}\;\Gamma_{b} \end{array}$$

(18i) $$\begin{array}{cc} T_{\delta }n_{\delta \beta }=T_{\beta }n_{\delta \beta } & \;\mathrm{on}\;\Gamma_{\delta \beta }\; \end{array}$$

(18j) $$\begin{array}{cc} \;\;u_{\delta }=u_{\beta } & \;\mathrm{on}\;\Gamma_{\delta \beta } \end{array}$$

For all δ=1,…,N,andβ=1,…,N, the primes have been removed to simplify the notation. The non-dimensionalised constitutive laws (5a), (5b), and (8) are

(19a) $$\begin{array}{cc} T_{f}=-pI+\xi (v)\; & \end{array}$$

(19b) $$\begin{array}{cc} \;T_{\delta }=C_{\delta }\xi \left(u_{\delta }\right) & \end{array}$$

(19c) $$\begin{array}{cc} T_{\beta }=C_{\beta }\xi \left(u_{\beta }\right), & \end{array}$$

and using these in the balance Equations (15a)–(15c) gives

(20a) $$\begin{array}{cc} \;\;\;\nabla^{2}v=\nabla p\; & \;\mathrm{in}\;\Omega_{f} \end{array}$$

(20b) $$\begin{array}{cc} \nabla \cdot (C_{\delta }\xi \left(u_{\delta }\right))=0\; & \;\mathrm{in}\;\Omega_{\delta } \end{array}$$

(20c) $$\begin{array}{cc} \nabla \cdot (C_{\beta }\xi \left(u_{\beta }\right))=0\; & \;\mathrm{in}\;\Omega_{b}. \end{array}$$

We are now ready to introduce the asymptotic homogenization method that will upscale the non-dimensional FSI problem (18a)–(20c) to macroscale governing equations by making the assumption that the two scales (micro and macro) are well separated.

### 3.2. The Asymptotic Homogenization Method

We now introduce the rules and assumptions associated with the asymptotic homogenization method which are then used to obtain the macroscale model from the FSI problem (18a)–(20c). We begin by making the assumption that the microscale length, which we denote by *d*, is much smaller than the average size of the viscoelastic material which has length *L*. That is,

(21) $$\epsilon =\frac{d}{L}\ll 1.$$

We require a microscale spatial variable which will describe how each field varies on the microscale, that is

(22) $$y=\frac{x}{\epsilon }.$$

We have two spatial variables x and y and it is assumed that these are formally independent, with x representing the macroscale and y the microscale. We have that the gradient operator will also transform

(23) $$\nabla \to \nabla_{x}+\frac{1}{\epsilon }\nabla_{y}.$$

We require that all the fields in (18a)–(20c) are functions of both spatial variables and have a power series in ϵ representation, i.e.,

(24) $$\varphi^{\epsilon }\left(x,y,t\right)=\sum_{l=0}^{\infty }\varphi^{\left(l\right)}\left(x,y,t\right)\epsilon^{l},$$

where φ is a generic field occurring in our analysis.

**Figure 2:**
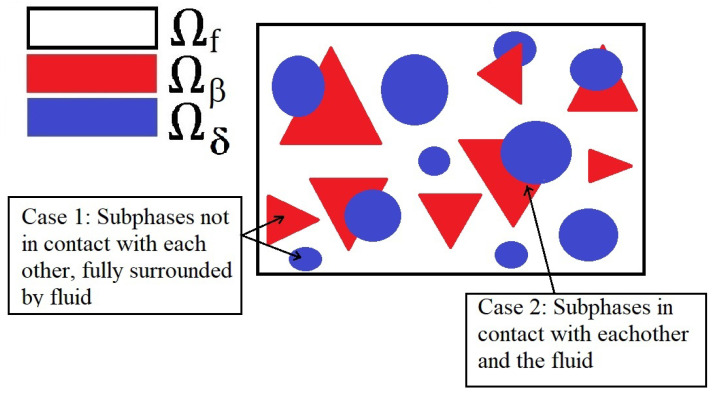
A 2D schematic highlighting a single periodic cell that creates our viscoelastic material. This is exactly a small piece of the the entire domain Ω. We have the fluid surrounding the solid phases is shown in white and the two different families of subphases are shown in red and blue. We see that each of the subphases $\Omega_{\delta }$ and $\Omega_{\beta }$ for δ=1,…,N,β=1,…,N, can be in contact with each other and the fluid or completely surrounded by fluid.

**Remark** **3** (Microscale periodicity)**.** *To simplify the analysis in this work, we restrict our attention to a single subset of the domain which we call the periodic cell. This periodic cell may have a variety of different subphases, each of which can have a different geometry and elastic properties, this is depicted in Figure 2. For this to be possible, we make the assumption that every field $\varphi^{\left(l\right)}$ arising in (18a)–(20c) is* **y***-periodic. By making this assumption, we can solve the microscale differential problems arising from using the asymptotic homogenization method on just a finite subset of the material. This assumption need not be made and the analysis can be carried using a less strict assumption, i.e., the local boundedness of fields. This approach, however, only allows us to determine the functional form of the macroscale equations and the model coefficients are to be obtained by solving microscale problems on the whole microstructure of the material. This makes solving the model very computationally expensive when using the local boundedness of fields approach in comparison to microscale periodicity. Some examples of this are found in [17,40,41].*

**Remark** **4** (Macroscopic uniformity)**.** *It is clear that the microscale geometry can differ depending on the macroscale point considered. This dependence is in general neglected in most works. We will make the assumption that the viscoelastic material is macroscopically uniform. That is, we assume the microscale geometry does not change with macroscale variable x. By making this assumption, we have the following*

(25) $$\int_{\Omega }\nabla_{x}\cdot \left(•\right)dy=\nabla_{x}\cdot \int_{\Omega }\left(•\right)dy.$$

*In the case that Ω=Ω(x), then Equation (25) does not hold. This leads to an application of the generalized Reynolds’ transport theorem which can give rise to additional contributions on the macroscale; see, e.g., [39,40,42].*

**Figure 3:**
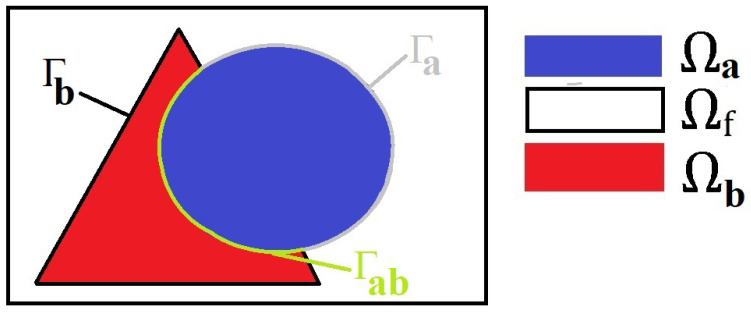
A 2D schematic of the periodic cell for our material. We are focusing on the geometry described in case 2 from Figure 2 where there are the two subphases (depicted in blue and red) in contact with each other and the fluid surrounding them which is shown in white. Here we also include the interfaces in our sketch. We have $\Gamma_{a}$ shown in grey between the inclusion a and the fluid, $\Gamma_{b}$ between the inclusion b and the fluid and $\Gamma_{\mathrm{ab}}$ shown in green is the solid–solid interface.

**Remark** **5** (Local Geometry)**.** *In the set up of the problem so far, we have considered a fluid with many embedded subphases that are interacting. For the sake of clarity, we assume that each family provides only one subphase for each periodic cell, as shown in Figure 3. This assumption does not affect the generality of the properties of the model. If a particular application requires a variety of subphases in the periodic cell, then this can easily be extended, as in [21]. Therefore, the indexes δ and β are not necessary and we can amend the notation. We identify the domain *Ω* with the corresponding periodic cell which comprises two solid subphases (one from each family) and fluid, with each of the phases denoted by $\Omega_{a}$, $\Omega_{b}$, and $\Omega_{f}$, respectively. We can also simplify the notation used for the interfaces between the different phases, so we have $\Gamma_{a}:=\partial \Omega_{a}\cap \partial \Omega_{f}$ between the fluid and the inclusion a, $\Gamma_{b}:=\partial \Omega_{b}\cap \partial \Omega_{f}$ is the interface between the fluid and the inclusion b, and $\Gamma_{\mathrm{ab}}:=\partial \Omega_{a}\cap \partial \Omega_{b}$, is the interface between the two solid phases. These interfaces have corresponding unit normal vectors $n_{a}$, $n_{b}$, and $n_{\mathrm{ab}}$.*

### 3.3. Deriving the Macroscale Model

We now apply the assumptions of the asymptotic homogenization method, i.e., (23) and (24), to Equations (18a)–(18j), accounting also for periodicity, to obtain

(26a) $$\begin{array}{cc} \nabla_{y}\cdot T_{a}^{\epsilon }+\epsilon \nabla_{x}\cdot T_{a}^{\epsilon }=0\; & \;\mathrm{in}\;\Omega_{a} \end{array}$$

(26b) $$\begin{array}{cc} \nabla_{y}\cdot T_{b}^{\epsilon }+\epsilon \nabla_{x}\cdot T_{b}^{\epsilon }=0\; & \;\mathrm{in}\;\Omega_{b} \end{array}$$

(26c) $$\begin{array}{cc} \nabla_{y}\cdot T_{f}^{\epsilon }+\epsilon \nabla_{x}\cdot T_{f}^{\epsilon }=0\; & \;\mathrm{in}\;\Omega_{f} \end{array}$$

(26d) $$\begin{array}{cc} \nabla_{y}\cdot v^{\epsilon }+\epsilon \nabla_{x}\cdot v^{\epsilon }=0\; & \;\mathrm{in}\;\Omega_{f} \end{array}$$

(26e) $$\begin{array}{cc} \;\;\;\;\;i\omega u_{a}^{\epsilon }=v^{\epsilon } & \;\mathrm{on}\;\Gamma_{a} \end{array}$$

(26f) $$\begin{array}{cc} \;\;\;\;\;i\omega u_{b}^{\epsilon }=v^{\epsilon } & \;\mathrm{on}\;\Gamma_{b} \end{array}$$

(26g) $$\begin{array}{cc} \;\;\;\;T_{f}^{\epsilon }n_{a}=T_{a}^{\epsilon }n_{a} & \;\mathrm{on}\;\Gamma_{a} \end{array}$$

(26h) $$\begin{array}{cc} \;\;\;\;T_{f}^{\epsilon }n_{b}=T_{b}^{\epsilon }n_{b} & \;\mathrm{on}\;\Gamma_{b} \end{array}$$

(26i) $$\begin{array}{cc} \;\;\;\;T_{a}^{\epsilon }n_{\mathrm{ab}}=T_{b}^{\epsilon }n_{\mathrm{ab}} & \;\mathrm{on}\;\Gamma_{\mathrm{ab}} \end{array}$$

(26j) $$\begin{array}{cc} \;\;\;\;\;\;u_{a}^{\epsilon }=u_{b}^{\epsilon } & \;\mathrm{on}\;\Gamma_{\mathrm{ab}}. \end{array}$$

The constitutive equations for the fluid and solid stress tensors under these assumptions are

(27a) $$\begin{array}{cc} \;\epsilon T_{f}^{\epsilon }=-\epsilon p^{\epsilon }I+\xi_{y}\left(v^{\epsilon }\right)+\epsilon \xi_{x}\left(v^{\epsilon }\right) & \end{array}$$

(27b) $$\begin{array}{cc} \;\;\;\epsilon T_{a}^{\epsilon }=C_{a}\xi_{y}\left(u_{a}^{\epsilon }\right)+\epsilon C_{a}\xi_{x}\left(u_{a}^{\epsilon }\right) & \end{array}$$

(27c) $$\begin{array}{c} \;\;\epsilon T_{b}^{\epsilon }=C_{b}\xi_{y}\left(u_{b}^{\epsilon }\right)+\epsilon C_{b}\xi_{x}\left(u_{b}^{\epsilon }\right), \end{array}$$

and the balance equations are

(28a) $$\begin{array}{cc} \; & \nabla_{y}\cdot \left(C_{a}\xi_{y}\left(u_{a}^{\epsilon }\right)\right)+\epsilon \nabla_{y}\cdot \left(C_{a}\xi_{x}\left(u_{a}^{\epsilon }\right)\right)+\epsilon \nabla_{x}\cdot \left(C_{a}\xi_{y}\left(u_{a}^{\epsilon }\right)\right)+\epsilon^{2}\nabla_{x}\cdot \left(C_{a}\xi_{x}\left(u_{a}^{\epsilon }\right)\right)\\ \; & =0\;\;\mathrm{in}\;\Omega_{a} \end{array}$$

(28b) $$\begin{array}{cc} \; & \;\nabla_{y}\cdot \left(C_{b}\xi_{y}\left(u_{b}^{\epsilon }\right)\right)+\epsilon \nabla_{y}\cdot \left(C_{b}\xi_{x}\left(u_{b}^{\epsilon }\right)\right)+\epsilon \nabla_{x}\cdot \left(C_{b}\xi_{y}\left(u_{b}^{\epsilon }\right)\right)+\epsilon^{2}\nabla_{x}\cdot \left(C_{b}\xi_{x}\left(u_{b}^{\epsilon }\right)\right)\\ \; & \;=0\;\;\mathrm{in}\;\Omega_{b} \end{array}$$

(28c) $$\begin{array}{cc} \; & \epsilon^{2}\nabla_{x}^{2}v^{\epsilon }+\epsilon \nabla_{x}\cdot \left(\nabla_{y}v^{\epsilon }\right)+\epsilon \nabla_{y}\cdot \left(\nabla_{x}v^{\epsilon }\right)+\nabla_{y}^{2}v^{\epsilon }\;\;\;\;\;\;\\ \; & =\epsilon \nabla_{y}p^{\epsilon }+\epsilon^{2}\nabla_{x}p^{\epsilon }\;\;\mathrm{in}\;\Omega_{f}\;\;\;\;\;\; \end{array}$$

Since all the fields can be written in terms of a power series of the type (24) in (26a)–(28c), we can equate the coefficients of $\epsilon^{l}$ for l=0,1,…. These obtained equations are then used to derive the macroscale model for the material. The model will be expressed in terms of relevant leading order fields. For any terms in the model that retain a dependence on the microscale variable y, we apply the integral average. This average is defined as

(29) $${\langle \varphi \rangle }_{i}:=\frac{1}{\left|\Omega \right|}\int_{\Omega_{i}}\varphi \left(x,y,t\right)dy\;i=f,a,b$$

where φ is a generic field and |Ω| is the volume of the domain and we note that $\left|\Omega |\;=\;\right|\Omega_{f}\left|\;+\;\right|\Omega_{a}\left|\;+\;\right|\Omega_{b}|$. Due to the assumption of y-periodicity, the integral average can be performed over one representative cell. Therefore, we can say that (29) represents a cell average.

We begin by equating coefficients of $\epsilon^{0}$ in (26a)–(26j) to obtain

(30a) $$\begin{array}{cc} \nabla_{y}\cdot T_{a}^{\left(0\right)}=0 & \;\;\mathrm{in}\;\Omega_{a}, \end{array}$$

(30b) $$\begin{array}{cc} \nabla_{y}\cdot T_{b}^{\left(0\right)}=0 & \;\;\mathrm{in}\;\Omega_{b}, \end{array}$$

(30c) $$\begin{array}{cc} \nabla_{y}\cdot {T_{f}}^{\left(0\right)}=0 & \;\;\mathrm{in}\;\Omega_{f}, \end{array}$$

(30d) $$\begin{array}{cc} \nabla_{y}\cdot v^{\left(0\right)}=0 & \;\;\mathrm{in}\;\Omega_{f}, \end{array}$$

(30e) $$\begin{array}{cc} i\omega u_{a}^{\left(0\right)}=v^{\left(0\right)} & \;\;\mathrm{on}\;\Gamma_{a}, \end{array}$$

(30f) $$\begin{array}{cc} i\omega u_{b}^{\left(0\right)}=v^{\left(0\right)} & \;\;\mathrm{on}\;\Gamma_{b}, \end{array}$$

(30g) $$\begin{array}{cc} T_{f}^{\left(0\right)}n_{a}=T_{a}^{\left(0\right)}n_{a} & \;\;\mathrm{on}\;\Gamma_{a}, \end{array}$$

(30h) $$\begin{array}{cc} T_{f}^{\left(0\right)}n_{b}=T_{b}^{\left(0\right)}n_{b} & \;\;\mathrm{on}\;\Gamma_{b}, \end{array}$$

(30i) $$\begin{array}{cc} T_{a}^{\left(0\right)}n_{\mathrm{ab}}=T_{b}^{\left(0\right)}n_{\mathrm{ab}} & \;\;\mathrm{on}\;\Gamma_{\mathrm{ab}}, \end{array}$$

(30j) $$\begin{array}{cc} \;u_{a}^{\left(0\right)}=u_{b}^{\left(0\right)} & \;\mathrm{on}\;\Gamma_{\mathrm{ab}}. \end{array}$$

The $\epsilon^{0}$ coefficients of the constitutive Equations (27a)–(27c) read

(31a) $$\begin{array}{cc} \;\xi_{y}\left(v^{\left(0\right)}\right)=0 & \;\mathrm{in}\;\Omega_{f}, \end{array}$$

(31b) $$\begin{array}{cc} C_{a}\xi_{y}\left(u_{a}^{\left(0\right)}\right)=0 & \;\;\mathrm{in}\;\Omega_{a}, \end{array}$$

(31c) $$\begin{array}{cc} C_{b}\xi_{y}\left(u_{b}^{\left(0\right)}\right)=0 & \;\;\mathrm{in}\;\Omega_{b}, \end{array}$$

and the balance Equations (28a)–(28c) have coefficients of $\epsilon^{0}$

(32a) $$\begin{array}{cc} \nabla_{y}\cdot \left(C_{a}\nabla_{y}\left(u_{a}^{\left(0\right)}\right)\right)=0 & \;\;\mathrm{in}\;\Omega_{a}, \end{array}$$

(32b) $$\begin{array}{cc} \nabla_{y}\cdot \left(C_{b}\nabla_{y}\left(u_{b}^{\left(0\right)}\right)\right)=0 & \;\;\mathrm{in}\;\Omega_{b}, \end{array}$$

(32c) $$\begin{array}{cc} \;\nabla_{y}^{2}v^{\left(0\right)}=0 & \;\mathrm{in}\;\Omega_{f}. \end{array}$$

We now similarly consider the coefficients of $\epsilon^{1}$ in (26a)–(26j) which leads to

(33a) $$\begin{array}{cc} \nabla_{y}\cdot T_{a}^{\left(1\right)}+\nabla_{x}\cdot T_{a}^{\left(0\right)}=0\; & \;\mathrm{in}\;\Omega_{a}, \end{array}$$

(33b) $$\begin{array}{cc} \nabla_{y}\cdot T_{b}^{\left(1\right)}+\nabla_{x}\cdot T_{b}^{\left(0\right)}=0\; & \;\mathrm{in}\;\Omega_{b}, \end{array}$$

(33c) $$\begin{array}{cc} \nabla_{y}\cdot {T_{f}}^{\left(1\right)}+\nabla_{x}\cdot {T_{f}}^{\left(0\right)}=0\; & \;\mathrm{in}\;\Omega_{f}, \end{array}$$

(33d) $$\begin{array}{cc} \nabla_{y}\cdot v^{\left(1\right)}+\nabla_{x}\cdot v^{\left(0\right)}=0\; & \;\mathrm{in}\;\Omega_{f}, \end{array}$$

(33e) $$\begin{array}{cc} \;i\omega u_{a}^{\left(1\right)}=v^{\left(1\right)} & \;\mathrm{on}\;\Gamma_{a}, \end{array}$$

(33f) $$\begin{array}{cc} \;i\omega u_{b}^{\left(1\right)}=v^{\left(1\right)} & \;\mathrm{on}\;\Gamma_{b}, \end{array}$$

(33g) $$\begin{array}{cc} \;T_{f}^{\left(1\right)}n_{a}=T_{a}^{\left(1\right)}n_{a} & \;\mathrm{on}\;\Gamma_{a}, \end{array}$$

(33h) $$\begin{array}{cc} \;T_{f}^{\left(1\right)}n_{b}=T_{b}^{\left(1\right)}n_{b} & \;\mathrm{on}\;\Gamma_{b}, \end{array}$$

(33i) $$\begin{array}{cc} \;T_{a}^{\left(1\right)}n_{\mathrm{ab}}=T_{b}^{\left(1\right)}n_{\mathrm{ab}} & \;\mathrm{on}\;\Gamma_{\mathrm{ab}}, \end{array}$$

(33j) $$\begin{array}{cc} \;u_{a}^{\left(1\right)}=u_{b}^{\left(1\right)} & \;\mathrm{on}\;\Gamma_{\mathrm{ab}}. \end{array}$$

Then, the constitutive Equations (27a)–(27c) have coefficients of $\epsilon^{1}$ given by

(34a) $$\begin{array}{cc} {T_{f}}^{\left(0\right)}=-p^{\left(0\right)}I+\xi_{y}\left(v^{\left(1\right)}\right)+\xi_{x}\left(v^{\left(0\right)}\right) & \;\mathrm{in}\;\Omega_{f}, \end{array}$$

(34b) $$\begin{array}{cc} \;T_{a}^{\left(0\right)}=C_{a}\xi_{y}\left(u_{a}^{\left(1\right)}\right)+C_{a}\xi_{x}\left(u_{a}^{\left(0\right)}\right) & \;\mathrm{in}\;\Omega_{a}, \end{array}$$

(34c) $$\begin{array}{cc} \;T_{b}^{\left(0\right)}=C_{b}\xi_{y}\left(u_{b}^{\left(1\right)}\right)+C_{b}\xi_{x}\left(u_{b}^{\left(0\right)}\right) & \;\mathrm{in}\;\Omega_{b}, \end{array}$$

and finally the balance Equations (28a)–(28c) have coefficients of $\epsilon^{1}$ given by

(35a) $$\begin{array}{cc} \nabla_{y}\cdot \left(C_{a}\xi_{y}\left(u_{a}^{\left(1\right)}\right)\right)+\nabla_{y}\cdot \left(C_{a}\xi_{x}\left(u_{a}^{\left(0\right)}\right)\right)+\nabla_{x}\cdot \left(C_{a}\xi_{y}\left(u_{a}^{\left(0\right)}\right)\right)=0 & \;\mathrm{in}\;\Omega_{a}, \end{array}$$

(35b) $$\begin{array}{cc} \nabla_{y}\cdot \left(C_{b}\xi_{y}\left(u_{b}^{\left(1\right)}\right)\right)+\nabla_{y}\cdot \left(C_{b}\xi_{x}\left(u_{b}^{\left(0\right)}\right)\right)+\nabla_{x}\cdot \left(C_{b}\xi_{y}\left(u_{b}^{\left(0\right)}\right)\right)=0 & \;\mathrm{in}\;\Omega_{b}, \end{array}$$

(35c) $$\begin{array}{cc} \;\nabla_{y}^{2}v^{\left(1\right)}+\nabla_{x}\cdot \left(\nabla_{y}v^{\left(0\right)}\right)+\nabla_{y}\cdot \left(\nabla_{x}v^{\left(0\right)}\right)=\nabla_{y}p^{\left(0\right)}\; & \;\mathrm{in}\;\Omega_{f}. \end{array}$$

We have from (31b), (31c), (32a) and (32b) and the periodicity conditions that $u_{a}^{\left(0\right)}$ and $u_{b}^{\left(0\right)}$ (the leading order solid displacements) are independent of the microscale variable y. We can also then describe these as rigid body motions and therefore we can write

(36a) $$\begin{array}{cc} u_{a}^{\left(0\right)}= & u_{a}^{\left(0\right)}\left(x\right) \end{array}$$

(36b) $$\begin{array}{cc} u_{b}^{\left(0\right)}= & u_{b}^{\left(0\right)}\left(x\right). \end{array}$$

Due to the continuity of elastic displacements $u_{a}^{\left(0\right)}=u_{b}^{\left(0\right)}$ on $\Gamma_{\mathrm{ab}}$ given by (30j), we can write

(37) $$u^{\left(0\right)}=u_{a}^{\left(0\right)}=u_{b}^{\left(0\right)},$$

and we use this in the remainder of this manuscript. We can also see from (30e) and (30f) that

(38a) $$\begin{array}{c} i\omega u_{a}^{\left(0\right)}=v^{\left(0\right)}, \end{array}$$

(38b) $$\begin{array}{c} i\omega u_{b}^{\left(0\right)}=v^{\left(0\right)}, \end{array}$$

this means that

(39) $$v^{\left(0\right)}=v^{\left(0\right)}\left(x\right),$$

and then using (37), we have that

(40) $$v^{\left(0\right)}=i\omega u^{\left(0\right)}.$$

### 3.4. Microscale Problem

We now wish to form a problem from the equations that we have just derived in the previous section. We will use conditions (36a), (36b), (37) and (39) and we can take balance Equations (35a)–(35c), we have incompressibility constraint (33d), continuities of velocities (33e), (33f), continuity of stresses (30g)–(30i), with leading order stresses from (34a)–(34c) and continuity of displacements (33j), to form the problem the problem

(41a) $$\begin{array}{cc} \;\nabla_{y}^{2}v^{\left(1\right)}-\nabla_{y}p^{\left(0\right)}=0 & \;\;\mathrm{in}\;\Omega_{f} \end{array}$$

(41b) $$\begin{array}{cc} \;\nabla_{y}\cdot v^{\left(1\right)}+\nabla_{x}\cdot v^{\left(0\right)}=0\; & \;\;\mathrm{in}\;\Omega_{f} \end{array}$$

(41c) $$\begin{array}{cc} \;v^{\left(1\right)}=i\omega u_{a}^{\left(1\right)} & \;\;\mathrm{on}\;\Gamma_{a} \end{array}$$

(41d) $$\begin{array}{cc} \;v^{\left(1\right)}=i\omega u_{b}^{\left(1\right)} & \;\;\mathrm{on}\;\Gamma_{b} \end{array}$$

(41e) $$\begin{array}{cc} \;\nabla_{y}\cdot \left(C_{a}\xi_{y}\left(u_{a}^{\left(1\right)}\right)\right)+\nabla_{y}\cdot \left(C_{a}\xi_{x}\left(u^{\left(0\right)}\right)\right)=0 & \;\;\mathrm{in}\;\Omega_{a} \end{array}$$

(41f) $$\begin{array}{cc} \;\nabla_{y}\cdot \left(C_{b}\xi_{y}\left(u_{b}^{\left(1\right)}\right)\right)+\nabla_{y}\cdot \left(C_{b}\xi_{x}\left(u^{\left(0\right)}\right)\right)=0 & \;\;\mathrm{in}\;\Omega_{b} \end{array}$$

(41g) $$\begin{array}{cc} \left(C_{a}\xi_{y}\left(u_{a}^{\left(1\right)}\right)+C_{a}\xi_{x}\left(u_{a}^{\left(0\right)}\right)\right)n_{a}=\left(-p^{\left(0\right)}I+\xi_{x}\left(v^{\left(0\right)}\right)+\xi_{y}\left(v^{\left(1\right)}\right)\right)n_{a} & \;\;\mathrm{on}\;\Gamma_{a} \end{array}$$

(41h) $$\begin{array}{cc} \left(C_{b}\xi_{y}\left(u_{b}^{\left(1\right)}\right)+C_{b}\xi_{x}\left(u_{b}^{\left(0\right)}\right)\right)n_{b}=\left(-p^{\left(0\right)}I+\xi_{x}\left(v^{\left(0\right)}\right)+\xi_{y}\left(v^{\left(1\right)}\right)\right)n_{b} & \;\;\mathrm{on}\;\Gamma_{b} \end{array}$$

(41i) $$\begin{array}{cc} \;C_{a}\xi_{y}\left(u_{a}^{\left(1\right)}\right)n_{\mathrm{ab}}-C_{b}\xi_{y}\left(u_{b}^{\left(1\right)}\right)n_{\mathrm{ab}}=\left(C_{b}-C_{a}\right)\xi_{x}\left(u^{\left(0\right)}\right)n_{\mathrm{ab}} & \;\;\mathrm{on}\;\Gamma_{\mathrm{ab}} \end{array}$$

(41j) $$\begin{array}{cc} \;u_{a}^{\left(1\right)}=u_{b}^{\left(1\right)} & \;\;\mathrm{on}\;\Gamma_{\mathrm{ab}} \end{array}$$

Since we have condition (40), we can replace the $v^{\left(0\right)}$ terms in Equations (41b), (41g) and (41h) with $i\omega u^{\left(0\right)}$. That is,

(42a) $$\begin{array}{cc} \;\nabla_{y}^{2}v^{\left(1\right)}-\nabla_{y}p^{\left(0\right)}=0 & \;\;\mathrm{in}\;\Omega_{f} \end{array}$$

(42b) $$\begin{array}{cc} \;\nabla_{y}\cdot v^{\left(1\right)}+i\omega \nabla_{x}\cdot u^{\left(0\right)}=0\; & \;\;\mathrm{in}\;\Omega_{f} \end{array}$$

(42c) $$\begin{array}{cc} \;v^{\left(1\right)}=i\omega u_{a}^{\left(1\right)} & \;\;\mathrm{on}\;\Gamma_{a} \end{array}$$

(42d) $$\begin{array}{cc} \;v^{\left(1\right)}=i\omega u_{b}^{\left(1\right)} & \;\;\mathrm{on}\;\Gamma_{b} \end{array}$$

(42e) $$\begin{array}{cc} \;\nabla_{y}\cdot \left(C_{a}\xi_{y}\left(u_{a}^{\left(1\right)}\right)\right)+\nabla_{y}\cdot \left(C_{a}\xi_{x}\left(u^{\left(0\right)}\right)\right)=0 & \;\;\mathrm{in}\;\Omega_{a} \end{array}$$

(42f) $$\begin{array}{cc} \;\nabla_{y}\cdot \left(C_{b}\xi_{y}\left(u_{b}^{\left(1\right)}\right)\right)+\nabla_{y}\cdot \left(C_{b}\xi_{x}\left(u^{\left(0\right)}\right)\right)=0 & \;\;\mathrm{in}\;\Omega_{b} \end{array}$$

(42g) $$\begin{array}{cc} \left(C_{a}\xi_{y}\left(u_{a}^{\left(1\right)}\right)+C_{a}\xi_{x}\left(u_{a}^{\left(0\right)}\right)\right)n_{a}=\left(-p^{\left(0\right)}I+i\omega \xi_{x}\left(u^{\left(0\right)}\right)+\xi_{y}\left(v^{\left(1\right)}\right)\right)n_{a} & \;\;\mathrm{on}\;\Gamma_{a} \end{array}$$

(42h) $$\begin{array}{cc} \left(C_{b}\xi_{y}\left(u_{b}^{\left(1\right)}\right)+C_{b}\xi_{x}\left(u_{b}^{\left(0\right)}\right)\right)n_{b}=\left(-p^{\left(0\right)}I+i\omega \xi_{x}\left(u^{\left(0\right)}\right)+\xi_{y}\left(v^{\left(1\right)}\right)\right)n_{b} & \;\;\mathrm{on}\;\Gamma_{b} \end{array}$$

(42i) $$\begin{array}{cc} \;C_{a}\xi_{y}\left(u_{a}^{\left(1\right)}\right)n_{\mathrm{ab}}-C_{b}\xi_{y}\left(u_{b}^{\left(1\right)}\right)n_{\mathrm{ab}}=\left(C_{b}-C_{a}\right)\xi_{x}\left(u^{\left(0\right)}\right)n_{\mathrm{ab}} & \;\;\mathrm{on}\;\Gamma_{\mathrm{ab}} \end{array}$$

(42j) $$\begin{array}{cc} \;u_{a}^{\left(1\right)}=u_{b}^{\left(1\right)} & \;\;\mathrm{on}\;\Gamma_{\mathrm{ab}} \end{array}$$

We now can exploit the linearity of the system (41a)–(41j) to write the following ansatz

(43a) $$\begin{array}{cc} \; & v^{\left(1\right)}=i\omega A\xi_{x}u^{\left(0\right)},\; \end{array}$$

(43b) $$\begin{array}{cc} \; & u_{a}^{\left(1\right)}=B_{a}\xi_{x}u^{\left(0\right)},\; \end{array}$$

(43c) $$\begin{array}{cc} \; & u_{b}^{\left(1\right)}=B_{b}\xi_{x}u^{\left(0\right)},\; \end{array}$$

(43d) $$\begin{array}{cc} \; & p^{\left(0\right)}=i\omega P:\xi_{x}u^{\left(0\right)}=\mathrm{Tr}\left(i\omega P\xi_{x}u^{\left(0\right)}\right), \end{array}$$

where A, $B_{a}$ and $B_{b}$ are third rank tensors and P is a second rank tensor. The auxiliary fields A, $B_{a}$, $B_{b}$ and P solve the following cell problem

(44a) $$\begin{array}{cc} \;\nabla_{y}^{2}A-\nabla_{y}P=0 & \end{array}$$

(44b) $$\begin{array}{cc} \;\nabla_{y}\cdot A^{T}+I=0\; & \end{array}$$

(44c) $$\begin{array}{cc} \;A=B_{a} & \end{array}$$

(44d) $$\begin{array}{cc} \;A=B_{b} & \end{array}$$

(44e) $$\begin{array}{cc} \;\nabla_{y}\cdot \left(C_{a}\xi_{y}\left(B_{a}\right)\right)+\nabla_{y}\cdot C_{a}=0 & \end{array}$$

(44f) $$\begin{array}{cc} \;\nabla_{y}\cdot \left(C_{b}\xi_{y}\left(B_{b}\right)\right)+\nabla_{y}\cdot C_{b}=0 & \end{array}$$

(44g) $$\begin{array}{cc} \left(C_{a}\xi_{y}\left(B_{a}\right)+C_{a}\right)n_{a}=\left(-i\omega I⊗P+i\omega I+i\omega \xi_{y}A\right)n_{a} & \end{array}$$

(44h) $$\begin{array}{cc} \left(C_{b}\xi_{y}\left(B_{b}\right)+C_{b}\right)n_{a}=\left(-i\omega I⊗P+i\omega I+i\omega \xi_{y}A\right)n_{b} & \end{array}$$

(44i) $$\begin{array}{cc} \;(C_{a}\xi_{y}\left(B_{a}\right)-C_{b}\xi_{y}\left(B_{b}\right))n_{\mathrm{ab}}=\left(C_{b}-C_{a}\right)n_{\mathrm{ab}} & \end{array}$$

(44j) $$\begin{array}{cc} \;B_{a}=B_{b} & \end{array}$$

where we can define $I=\delta_{ij}\delta_{kl}$ as a fourth rank identity tensor.

Problem (44a)–(44j) is to be solved on the periodic cell and will also require periodic conditions on the external boundaries of Ω. In order to obtain a unique solution, we place one further condition on the auxiliary variables A, $B_{a}$, $B_{b}$ and P, that is

(45) $${\langle A\rangle }_{f}=0,\;{\langle B_{a}\rangle }_{a}=0,\;{\langle B_{b}\rangle }_{b}=0,\;{\langle P\rangle }_{f}=0$$

### 3.5. Balance Equation on the Macroscale

We must derive the macroscale balance equation. We apply the integral averages to Equations (33a)–(33c) and sum up to obtain

(46) $$\begin{array}{cc} \; & \int_{\Omega_{a}}\nabla_{y}\cdot T_{a}^{\left(1\right)}dy+\int_{\Omega_{b}}\nabla_{y}\cdot T_{b}^{\left(1\right)}dy+\int_{\Omega_{f}}\nabla_{y}\cdot {T_{f}}^{\left(1\right)}dy+\\ \; & \int_{\Omega_{a}}\nabla_{x}\cdot T_{a}^{\left(0\right)}dy+\int_{\Omega_{b}}\nabla_{x}\cdot T_{b}^{\left(0\right)}dy+\int_{\Omega_{f}}\nabla_{x}\cdot {T_{f}}^{\left(0\right)}dy=0. \end{array}$$

The divergence theorem can be applied to the first three integrals and we can use macroscopic uniformity condition (25) to rearrange the last three integrals so that we obtain

(47) $$\begin{array}{cc} \; & \int_{\partial \Omega_{a}\setminus \Gamma_{a}\cup \Gamma_{\mathrm{ab}}}T_{a}^{\left(1\right)}n_{\Omega_{a}\setminus \Gamma_{a}\cup \Gamma_{\mathrm{ab}}}\mathrm{dS}+\int_{\Gamma_{a}}T_{a}^{\left(1\right)}n_{a}\mathrm{dS}-\int_{\Gamma_{\mathrm{ab}}}T_{a}^{\left(1\right)}n_{\mathrm{ab}}\mathrm{dS}+\\ \; & \int_{\partial \Omega_{b}\setminus \Gamma_{b}\cup \Gamma_{\mathrm{ab}}}T_{b}^{\left(1\right)}n_{\Omega_{b}\setminus \Gamma_{b}\cup \Gamma_{\mathrm{ab}}}\mathrm{dS}+\int_{\Gamma_{b}}T_{b}^{\left(1\right)}n_{b}\mathrm{dS}+\int_{\Gamma_{\mathrm{ab}}}T_{b}^{\left(1\right)}n_{\mathrm{ab}}\mathrm{dS}+\\ \; & \int_{\partial \Omega_{f}\setminus \Gamma_{a}\cup \Gamma_{b}}T_{f}^{\left(1\right)}n_{\Omega_{f}\setminus \Gamma_{a}\cup \Gamma_{b}}\mathrm{dS}-\int_{\Gamma_{b}}T_{f}^{\left(1\right)}n_{b}\mathrm{dS}-\int_{\Gamma_{a}}T_{f}^{\left(1\right)}n_{a}\mathrm{dS}+\\ \; & \nabla_{x}\cdot \int_{\Omega_{a}}T_{a}^{\left(0\right)}dy+\nabla_{x}\cdot \int_{\Omega_{b}}T_{b}^{\left(0\right)}dy+\nabla_{x}\cdot \int_{\Omega_{f}}{T_{f}}^{\left(0\right)}dy=0, \end{array}$$

where the vectors $n_{a}$, $n_{b}$, $n_{\mathrm{ab}}$, $n_{\Omega_{I}\setminus \Gamma_{a}\cup \Gamma_{\mathrm{ab}}}$, $n_{\Omega_{b}\setminus \Gamma_{b}\cup \Gamma_{\mathrm{ab}}}$ and $n_{\Omega_{f}\setminus \Gamma_{a}\cup \Gamma_{b}}$ are the unit normals to the interfaces $\Gamma_{a}$, $\Gamma_{b}$, $\Gamma_{\mathrm{ab}}$, $\partial \Omega_{a}\setminus \Gamma_{a}\cup \Gamma_{\mathrm{ab}}$, $\partial \Omega_{b}\setminus \Gamma_{b}\cup \Gamma_{\mathrm{ab}}$ and $\partial \Omega_{f}\setminus \Gamma_{a}\cup \Gamma_{b}$. Due to y-periodicity, we can cancel the terms that arise on the external boundaries of the phases $\Omega_{a}$, $\Omega_{b}$ and $\Omega_{f}$. This gives

(48) $$\begin{array}{cc} \; & \int_{\Gamma_{a}}T_{a}^{\left(1\right)}n_{a}\mathrm{dS}+\int_{\Gamma_{b}}T_{b}^{\left(1\right)}n_{b}\mathrm{dS}-\int_{\Gamma_{a}}T_{f}^{\left(1\right)}n_{a}\mathrm{dS}-\\ \; & \int_{\Gamma_{b}}T_{f}^{\left(1\right)}n_{b}\mathrm{dS}-\int_{\Gamma_{\mathrm{ab}}}T_{a}^{\left(1\right)}n_{\mathrm{ab}}\mathrm{dS}+\int_{\Gamma_{\mathrm{ab}}}T_{b}^{\left(1\right)}n_{\mathrm{ab}}\mathrm{dS}+\\ \; & \nabla_{x}\cdot \int_{\Omega_{I}}T_{a}^{\left(0\right)}dy+\nabla_{x}\cdot \int_{\Omega_{b}}T_{b}^{\left(0\right)}dy+\nabla_{x}\cdot \int_{\Omega_{f}}{T_{f}}^{\left(0\right)}dy=0. \end{array}$$

The continuity of stresses interface conditions, Equations (33g)–(33i), can be used to cancel out the first six integrals in (48). This means that the remaining terms can be written as

(49) $$\begin{array}{cc} \; & \nabla_{x}\cdot \left({\langle T_{a}^{\left(0\right)}\rangle }_{a}+{\langle T_{b}^{\left(0\right)}\rangle }_{b}+{\langle T_{f}^{\left(0\right)}\rangle }_{f}\right)=0. \end{array}$$

Our balance equation comprises the zero-th order solid stress tensors. Using Equations (34b) and (34c), we know that $u_{a}^{\left(1\right)}$ and $u_{b}^{\left(1\right)}$ appear in $T_{a}^{\left(0\right)}$ and $T_{b}^{\left(0\right)}$, respectively. Therefore, we write the leading order solid stresses as

(50) $$T_{a}^{\left(0\right)}=C_{a}M_{a}\xi_{x}\left(u^{\left(0\right)}\right)+C_{a}\xi_{x}\left(u^{\left(0\right)}\right)$$

and

(51) $$T_{b}^{\left(0\right)}=C_{b}M_{b}\xi_{x}\left(u^{\left(0\right)}\right)+C_{b}\xi_{x}\left(u^{\left(0\right)}\right)$$

making use of the notation

(52) $$\begin{array}{c} M_{a}:=\xi_{y}\left(B_{a}\right),\;M_{b}:=\xi_{y}\left(B_{b}\right). \end{array}$$

We also want to write down the zero-th order fluid stress (34a) since we have an expression for $v^{\left(1\right)}$

(53) $${T_{f}}^{\left(0\right)}=-p^{\left(0\right)}I+i\omega \left(L+I\right)\xi_{x}u^{\left(0\right)},$$

or equivalently

(54) $${T_{f}}^{\left(0\right)}=-p^{\left(0\right)}I+\left(L+I\right)\xi_{x}v^{\left(0\right)},$$

where we have defined

(55) $$L:=\xi_{y}A.$$

Adding (50), (51) and (53) and applying the integral average over each of the domains gives

(56) $$\begin{array}{cc} {\langle T_{a}^{\left(0\right)}\rangle }_{a}+{\langle T_{b}^{\left(0\right)}\rangle }_{b}+{\langle T_{f}^{\left(0\right)}\rangle }_{f}= & ({\langle C_{a}M_{a}+C_{a}\rangle }_{a}+{\langle C_{b}M_{b}+C_{b}\rangle }_{b})\xi_{x}\left(u^{\left(0\right)}\right)\\ \; & +{\langle \left(L+I-\left(\mathrm{Tr}P\right)I\right)\rangle }_{f}\xi_{x}\left(v^{\left(0\right)}\right) \end{array}$$

From (49) we know that

(57) $$\nabla_{x}\cdot T_{\mathrm{Eff}}=0$$

with

(58) $$T_{\mathrm{Eff}}={\langle T_{a}^{\left(0\right)}\rangle }_{a}+{\langle T_{b}^{\left(0\right)}\rangle }_{b}+{\langle T_{f}^{\left(0\right)}\rangle }_{f}$$

We are able to describe (57) and (58) as the averaged force balance equation for our material.

## 4. The Macroscale Governing Equations and Limit Cases

We now have derived the equations necessary to write the macroscale governing equations for a linear viscoelastic composite material. That is

(59a) $$\begin{array}{cc} \; & \nabla_{x}\cdot T_{\mathrm{Eff}}=0,\; \end{array}$$

(59b) $$\begin{array}{cc} \; & T_{\mathrm{Eff}}=\left({\langle C_{a}M_{a}+C_{a}\rangle }_{a}+{\langle C_{b}M_{b}+C_{b}\rangle }_{b}\right)\xi_{x}\left(u^{\left(0\right)}\right)+{\langle \left(L+I-\left(\mathrm{Tr}P\right)I\right)\rangle }_{f}\xi_{x}\left(v^{\left(0\right)}\right), \end{array}$$

where $u^{\left(0\right)}$ is the leading order solid displacement, $v^{\left(0\right)}$ is the leading order fluid velocity and $p^{\left(0\right)}$ is the leading order pressure. Equation (59a) is the balance equation with the new constitutive law for viscoelastic composites given by (59b). We can see that the viscoelastic constitutive law takes exactly the form that is expected of a Kelvin–Voigt viscoelastic material comprising first the elastic constitutive relationship and the the second part is the viscous part of the relation as in Kelvin–Voigt materials. The addition of the multiple elastic phases being encoded in our model influences the elastic part of our constitutive law.

The new model we derive has an important difference from previous formulations of standard viscoelastic materials. Our model has the ability to incorporate multiple elastic phases all interplaying with the fluid and each other, whereas previous viscoelastic formulations of this kind, such as [17], only consider one fluid and one solid phase. These additional interactions between the multiple phases can be extremely useful in physical systems where the solid component is rarely homogeneous. The ability to model heterogeneous materials comes from the fact that discrepancies in the elastic and mechanical properties of each phase are accounted for by the multiple elasticity tensors $C_{a}$ and $C_{b}$ as well as the tensors $M_{a}$ and $M_{b}$ which account for the differences in the elastic properties at different points in the microstructure. In this work, we propose the novel cell problem (44a)–(44j), this is the problem from which the model coefficients are determined. The cell problem is an extension to the problem found for viscoelastic materials in [17] and comprises some of the elastic problem associated with poroelastic composites [38]. This cell problem comprises both the fluid and solid equations in one problem and therefore we do not have the decoupling of the different phases as seen in poroelastic-type cell problems. The model as it stands can be described as a comprehensive framework for Kelvin–Voigt viscoelastic materials that comprise various elastic phases.

We now wish to understand how our macroscale model for viscoelastic composites (59a) and (59b) compares and can reduce to previous models in the literature. We consider the viscoelastic model derived via asymptotic homogenization in [17] which considers only one elastic phase and the fluid, and we consider the model for poroelastic composites by [38] which addresses the interaction of a porous matrix and and elastic phase where fluid flows in the pores which are also comparable in size to the distance between the inclusions.

**Remark** **6** (Comparison with Burridge and Keller [17])**.** *We now wish to compare the model we derived with the results of the [17] in the remark of effective viscoelasticity. There, the authors consider one elastic phase and one fluid phase and the interactions between them. We begin the comparison by rewriting the fluid stress ${T_{f}}^{\left(0\right)}$, from (53), using both the expression for $v^{\left(1\right)}$ and $p^{\left(0\right)}$. That is,*

(60) $${T_{f}}^{\left(0\right)}=i\omega \left(L+I-\left(\mathrm{Tr}P\right)I\right)\xi_{x}u^{\left(0\right)}.$$

*We can now use this version of the fluid stress in (56) to obtain*

(61) $$\begin{array}{cc} {\langle T_{a}^{\left(0\right)}\rangle }_{a}+{\langle T_{b}^{\left(0\right)}\rangle }_{b}+{\langle T_{f}^{\left(0\right)}\rangle }_{f} & =({\langle C_{a}M_{a}+C_{a}\rangle }_{a}+{\langle C_{b}M_{b}+C_{b}\rangle }_{b}\\ \; & +{\langle i\omega \left(L+I-\left(\mathrm{Tr}P\right)I\right)\rangle }_{f})\xi_{x}\left(u^{\left(0\right)}\right). \end{array}$$

*This is still the constitutive law of our model, it had just been presented in a different form. We then rewrite the macroscale model (59a) and (59b) as*

(62a) $$\begin{array}{cc} \; & \nabla_{x}\cdot T_{\mathrm{Eff}}=0,\; \end{array}$$

(62b) $$\begin{array}{cc} \; & T_{\mathrm{Eff}}=\left({\langle C_{a}M_{a}+C_{a}\rangle }_{a}+{\langle C_{b}M_{b}+C_{b}\rangle }_{b}+{\langle i\omega \left(L+I-\left(\mathrm{Tr}P\right)I\right)\rangle }_{f}\right)\xi_{x}\left(u^{\left(0\right)}\right). \end{array}$$

*The constitutive equation comprises the average of the stresses in both the solid domains I and II and fluid domain of the material. In [17], the constitutive law is written as the sum of the leading order stress in the solid and the leading order stress in the fluid. If we assumed we had indeed only one solid phase, our constitutive law will match that of [17]. That is,*

(63) $$T_{\mathrm{Eff}}=\left({\langle C_{a}M_{a}+C_{a}\rangle }_{a}+{\langle i\omega \left(L+I-\left(\mathrm{Tr}P\right)I\right)\rangle }_{f}\right)\xi_{x}\left(u^{\left(0\right)}\right),$$

*and we can identify $T_{\mathrm{Eff}}={\langle T_{a}^{\left(0\right)}\rangle }_{a}+{\langle T_{f}^{\left(0\right)}\rangle }_{f}$ with the notation $\bar{\tau }={\bar{\tau }}_{0}+{\bar{\sigma }}_{0}$ which has been used in [17]. In this work, we find explicit forms of the leading order stresses by proposing a solution to the problem (41a)–(41j). In [17], the suggested equations to form a linear dissipative problem are the same as those we have in (41a)–(41j), with the exception that we have two solid elastic phases and they consider only one. In their work, they do not explicitly solve this problem, but propose that the leading order stresses in the solid and fluid are proportional to the macroscale gradient of the leading order solid displacement $u^{\left(0\right)}$ as we have done here.*

**Remark** **7** (Comparison with Poroelastic Composites [38])**.** *We now can consider each of the differences between this model and that of [38] for poroelastic composites. In the work of [38], the authors consider a porous matrix with embedded elastic subphases that are in contact with the matrix and the fluid that flows in connected cylindrical pores. Due to the profile of the fluid flowing in the pores, [38] have that the fluid velocity scales by $v=\frac{Cd^{2}}{\mu }v^{\prime }$, where d is the radius of the pores, which is the standard scaling for Stokes’ flow in porous media. In this current work we have that the pores are much larger and therefore scale by $v=\frac{CL^{2}}{\mu }v^{\prime }$. These two different choices relate to the observed microstructure and result in the appearance of an $\epsilon^{2}$ coefficient in the fluid stress tensor and in the Stokes equation in porous media, but not in the case of viscoelastic media. This means that when applying the asymptotic homogenization method, the Stokes-type equation and the fluid stress tensor will have different orders and terms than those that we equate in this work. That is, the Stokes equation in [38] reads*

(64) $$\nabla_{y}^{2}v^{\left(0\right)}-\nabla_{y}p^{\left(1\right)}=\nabla_{x}p^{\left(0\right)},$$

*we can see that the orders of $\nabla_{y}^{2}v$ and $\nabla_{y}p$ are switched when compared with (41a) and [38] has the additional macroscale gradient of the pressure. The fluid stress of [38] is also different*

(65) $$T^{\left(0\right)}=-p^{\left(0\right)}I,$$

*when compared with (34a), and we see that there are no micro or macroscale gradients of the fluid velocity v.*

*Equations (42e), (42f), (42i) and (42j) appearing in the problem we derive are analogous to those that form part of the elastic problem in [38]. However, the difference in the scaling of the fluid stress leads to different continuity of stresses across the fluid–solid interfaces (42g) and (42h). That is, in [38], we have the continuity of stresses using (65), (34b) and (34c) instead of that found in (34a).*

*These small changes in the orders of the terms lead to the formation of only one linear dissipative problem in this work with no decoupling of the phases, rather than being able to separate the problem into a fluid problem and an elastic problem as in [38].*

## 5. Conclusions

In this work, we have presented a system of PDEs governing the effective mechanical behaviour of viscoelastic composites. Our constitutive law takes the form of that of a Kelvin–Voigt material, where we have the effective elasticity tensor applied to the strain plus the viscosity applied to the time derivative of the strain. Our structure comprises multiple solid fibres/subphases and a fluid phase, all of which are in contact with each other. This structure is widely applicable to many real world scenarios including modelling of human bones [3,4,8,9] and skin [5,6,7], as well as in many engineering and manufacturing problems such as biomimetic materials [43] and polymers [44,45].

We begin our analysis by formulating the quasi-static fluid–structure interaction problem describing the behaviour of two families of linear elastic inclusions/fibres interacting with an incompressible Newtonian fluid. We then have made the assumption that both the fluid and the elastic fibres/inclusions/subphases are clearly visible and distinct on the microscale, and also assumed that the macroscale represents the average size of material we are modelling. These two scales are distinct, and their ratio leads to a very small scale separation parameter. We then enforce this distinction in length scales to upscale the non-dimensionalised FSI problem via asymptotic homogenization. The new model derived in this way retains the important properties of the microstructure such as geometry and stiffness in its coefficients, which are determined via solving the presented novel periodic cell problem.

The new model that we derive comprises a balance equation and constitutive law. The viscoelastic constitutive law takes exactly the form that is expected of a viscoelastic material by comprising first the elastic constitutive relationship and second the viscous part of the relation. The addition of the multiple elastic phases in our model influences the elastic part of our constitutive law which reads like that of an elastic composite.

Our new model has an important distinction from formulations of previous viscoelastic models. That is, our model encodes multiple different elastic phases interplaying with each other and the fluid in comparison with some previous viscoelastic formulations where there is only one fluid and solid phase. Accounting for these additional interactions between the multiple phases can be of particular benefit to physical applications. The differences in elastic and mechanical properties between the phases are accounted for by the multiple elasticity tensors. The latter appear in the constitutive law and are accompanied by tensors accounting for the discrepancies in the elastic properties at different points in the microstructure. In this work, we propose the novel cell problem (44a)–(44j) from which the model coefficients are calculated. The cell problem is an extension to the problem found for viscoelastic materials in [17] and comprises the elastic problem associated with poroelastic composites. This cell problem comprises both the fluid and solid equations in one problem and therefore we do not have the decoupling of the different phases as seen in poroelastic-type cell problems.

The addition of linearised inertia and compressibility of fluid to our model would be straightforward. In this case, we would have a system similar to that found in [17]. These additions would also result in corresponding changes to the macroscale model. The effective balance equation that we present in our macroscale model would have the addition of linearised leading order inertia. The compressibility of the fluid would have an influence on the cell problem with the fluid bulk modulus appearing in the Stokes-type problem component.

The current model assumes that both families of elastic phases are anisotropic linear elastic materials. We could, however, extend the model to assume that these phases exhibit hyperelastic behaviour. To do this, we would use a method similar to that found in [31,46,47,48]. By having hyperelastic phases, we dramatically increase the complexity of the numerical simulations that are to be carried out to compute the model coefficients and finally the macroscale model solution. The additional complexity is due to the fact that the two length scales (macro and micro) remain coupled. In the literature, methods to address the remaining coupling of the scales are emerging, see [49,50]. We also note that the present work can be extended for soft hyperelastic electro-active [51] and magneto-active composites [52]. This extension could provide many interesting and exciting applications in modern actuators, soft robotics and biomedicine.

There are many future directions in which this work could be developed. A first step could be to consider the cell problems using the Fourier transform method and then applying the inverse transform to obtaining a formulation in the time domain, see, e.g., ref. [53]. This would lead to cell problems that are computationally feasible to solve that have been parameterised based on real-world data which could be from, for example, biological tissues. Numerical computations performed by finite element simulations have been used in [32] to study the homogenized properties of linear viscoelastic composite materials in three dimensions by means of a semi-analytical approach combined with the asymptotic homogenization method. Finally, our formulation only accounts for Kelvin–Voigt viscoelastic materials at the macroscale. An interesting development of the theory resides in considering more complex constitutive relationships for the individual phases in order to obtain a more general framework for more general viscoelastic behaviours, such as those described in [54].
